# Distinct brain and neurocognitive transformations after bariatric surgery: a pilot study

**DOI:** 10.3389/fnins.2024.1454284

**Published:** 2024-11-05

**Authors:** Bhaswati Roy, Mariana Thedim, Chiewlin Liew, Rajesh Kumar, Susana Vacas

**Affiliations:** ^1^Department of Anesthesiology and Perioperative Medicine, University of California, Los Angeles, Los Angeles, CA, United States; ^2^Department of Anesthesia, Critical Care and Pain Medicine, Massachusetts General Hospital, Harvard Medical School, Boston, MA, United States; ^3^Department of Radiology, University of California, Los Angeles, Los Angeles, CA, United States; ^4^Department of Bioengineering, University of California, Los Angeles, Los Angeles, CA, United States

**Keywords:** bariatric surgery, cognitive dysfunction, magnetic resonance imaging, neuropsychological tests, obesity, perioperative neurocognitive disorders, surgery, postoperative delirium

## Abstract

**Background:**

Obese patients have worse outcomes after surgery and are at increased risk for perioperative neurocognitive disorders (PND). Our aim was to detail the cognitive trajectories of patients undergoing bariatric surgery (BS) and map distinct structural brain changes using magnetic resonance imaging (MRI) to better understand the association between the vulnerable brain, surgery, and the arc of PND.

**Methods:**

Prospective pilot study with longitudinal comprehensive cognitive assessments and MRI were performed on obese patients scheduled for BS. We analyzed baseline cognitive function and high-resolution T1-/T2-weighted brain images on 19 obese patients [age, 54 (9) years, BMI, 40 (36, 42) kg m^−2^] and compared with 50 healthy control subjects [age, 52 (6) years; BMI, 25 (24, 27) kg m^−2^]. Patients were evaluated within five days of BS (baseline), immediately after (within 48h), and follow up at six months.

**Results:**

At baseline, obese patients had significant brain tissue changes seen in MRI and decreased cognitive scores compared to controls (MoCA 26 vs 28, *P* = 0.017). Surgery induced further gray matter volume and brain tissue changes along with reduced cognitive scores within the immediate postoperative period (MoCA 26 vs 24, *P* < 0.001). At six months, we observed reversal of brain alterations for most patients and a concomitant rebound of cognitive scores to patient’s baseline status.

**Conclusions:**

Bariatric surgery resulted in worsening of preexisting brain structural integrity and lower cognitive function for obese patients compared to baseline. These distinct brain lesions are consistent with specific domains of cognition. Most of these changes reverted to patient’s baseline condition within six months after surgery.

## 1 Introduction

Obesity is a massive and global health concern, raising the risk of death, cardiovascular disease, cancer, and dementia (Islami et al., 2019; Ma et al., 2020; Powell-Wiley et al., 2021). Brain alterations in overweight and obese populations are reflected in lower cognitive performance and diminished executive function (Prickett et al., 2015). In addition, obese patients have worse outcomes after surgery and are at increased risk for perioperative neurocognitive disorders (PND).

Although new drug therapies have gained traction around the world, bariatric surgery (BS) remains a viable intervention that can reverse the comorbidities associated with this condition (Yu et al., 2015). While weight loss has the potential to improve neurocognitive function of obese patients, the potential for harm in vulnerable brain areas for patients with surgery and anesthesia requires careful consideration. Patients who develop PND are at increased risk of functional and psychological health decline, progressive cognitive impairment, dementia, and ultimately death (Vacas et al., 2021; Vacas et al., 2022). Cognitive dysfunction in obese populations remains an actively researched topic that impacts the quality of life and mortality for millions of people worldwide (Burns et al., 2023; World Health Organization, 2024).

The complexity of cognitive changes after surgery and anesthesia is partly due to the heterogeneity of contributing factors and different etiologies. Neuroinflammation, dysregulation of the cholinergic anti-inflammatory pathway, abnormal response to perioperative stress, vascular abnormalities, changes in oxidative cellular metabolism, acceleration of undiagnosed neurocognitive decline or even newly discovered mechanisms, such as dysfunction of the glymphatic system, may have different but overlapping influences on a range of vulnerable populations (Mahanna-Gabrielli et al., 2019; Roy et al., 2024). Metainflammation, a chronic state of metabolic inflammation, that is usually observed in obese patients is linked with the underlying pathology of obesity-related comorbidities (Charles-Messance et al., 2020) and plays a role in the amplified and persistent cognitive decline observed after surgery (Feng et al., 2013; Gambus et al., 2015). Obese patients are both predisposed and more susceptible to PND, which greatly impacts short and long-term outcomes to brain health (Ma et al., 2020; Burns et al., 2023). For example, the physiologic changes that accompany obesity share similarities with the mechanisms involved with PND (Vacas et al., 2021). Importantly, BS patients offer an opportunity to better understand the underlying causes, mechanisms, morphology, and fluctuations in brain tissue after surgery and anesthesia-induced PND in at-risk populations because it offers a clear throughline from which we can trace the arc of PND. To put it differently, the positive benefits of the surgery, namely the loss of weight and diminishment of attendant comorbidities, complicate the understanding of cognitive recovery of obese patients after BS because the procedure that is meant to address the very problem that is directly causing the vulnerability in the first place is also placing the BS patients at higher risk for PND. Our aim was to untangle the alterations and variabilities in cognition and associated structural brain changes to better understand the relationship between the vulnerable brain and surgery. Therefore, we would expect that the arc of any changes we observe in both cognitive assessment and brain imaging would move in tandem along a timeline that is markedly more pronounced and amplified, both in sequence and dimension than we would observe otherwise. As a logical corollary, we focus on at-risk populations undergoing surgery to better gauge the specific brain areas involved, anatomical and volumetric alterations within, and neurological and biological mechanisms at play in its initiation and possible change of PND along with its possible variations, oscillations, and transformations at different timepoints in the perioperative period, which might aid in the conception and development of effective preventive and therapeutic strategies.

Cognitive assessments are crucial to tracking changes to the brain but do not provide morphological or functional regional brain health impacts. Magnetic resonance imaging (MRI) coupled with cognitive assessments can provide a valuable and nuanced picture of the neurocognitive changes in the vulnerable brain during the perioperative period (Browndyke et al., 2021; Hardcastle et al., 2019). Our previous studies showed that MRI and cognitive assessment can provide viable insights into brain changes after surgery (Roy et al., 2024).

The primary objective of this pilot study was to assess cognitive trajectories of patients submitted to bariatric surgery using MRI and cognitive assessments at baseline, < 48h after surgery, and with follow up after six months.

## 2 Materials and methods

This prospective cohort study received approval by the Institutional Review Board of the University of California Los Angeles on September 12, 2019, and was registered with ClinicalTrials.gov before enrolment of any participants. All subjects provided informed written consent before study involvement. This report follows STROBE checklist for observational studies.

### 2.1 Participants

We recruited patients aged 40 and older, scheduled for bariatric surgery, including laparoscopic sleeve gastrectomy and Roux-en-Y gastric bypass, under general anesthesia between December 2020 and April 2022 (BS group). Patients with cardiac, renal, or hepatic failure, vascular or neurologic disease, severe depression, excessive alcohol use, repeated use of opioids or other illicit substances, or body weight > 125 kg (scanner limitation) were excluded. To avoid confounders related to intraoperative management, all patients received the same consistent general anesthetic plan. Patients were monitored according to the American Society of Anesthesiologists guidelines, and current brain health recommendations (Berger et al., 2018) with continuous noninvasive blood pressure monitoring (Clearsight; Edwards Lifesciences, Irvine, CA) and four channel electroencephalogram (EEG) (SedLine^®^, Masimo, Irvine, CA). Adjunct medications, such as midazolam, ketamine, or dexmedetomidine were not administered.

**FIGURE 1:**
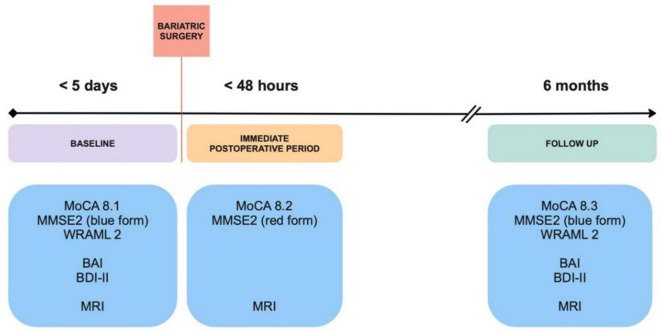
Study design. Obese patients scheduled for bariatric surgery were assessed for cognition and underwent imagining studies within five days before surgery, in the immediate postoperative period and follow up at 6 months. BAI, Beck Anxiety Inventory; BDI, Beck Depression Inventory; MMSE2, Mini-Mental State Examination 2nd edition; MoCA, Montreal Cognitive Assessment; MRI, Magnetic Resonance Imaging; WRAML2, Wide Range Assessment of Memory and Learning 2.

Patients were evaluated at three different time points: baseline (within five days before surgery), postoperatively (within 48h after surgery), and with follow up at six months (Figure 1). To establish acuity of baseline brain lesions seen in MRI, our control population was composed of healthy subjects with normal body mass index (BMI), without signs of neurological diseases and not taking medications that could affect brain function. This data was retrieved from an existing database and matched with the BS group for age and sex. Education level was recorded for all participants. Control subjects were not submitted to surgery and were evaluated once.

### 2.2 Neurocognitive assessment

All study subjects were submitted to comprehensive neurocognitive assessments by trained clinical team members. The tests were chosen on their ability to assess several memory domains, high sensitivity and specificity, and availability of alternative and equivalent versions to track deterioration over time.

Control subjects and BS patients’ cognition was assessed at baseline with the Montreal Cognitive Assessment (MoCA) version 8, a 30-point test with alternate forms, where a score equal to or above 26 is considered normal (Nasreddine et al., 2005). Subsequently, BS patients were evaluated with MoCA < 48h after surgery and with follow up after six months. The Mini-Mental State Examination 2^nd^ edition (MMSE2) also has alternate forms, with a maximum score of 90 points in the extended version (Lee et al., 2022). The three available versions of this assessment were conducted at all time points in the BS group. BS patients were also submitted to the Wide Range Assessment of Memory and Learning 2 (WRAML2) at baseline and six months after surgery. This broad-based memory battery provides a flexible measure of memory functioning and learning with a maximum general memory index of 114 points (Atkinson et al., 2008; Figure 1). Alternative and equivalent forms of the cognitive tests were used to reduce learning effects and test scores were normalized for age and education level.

As part of the neuropsychological assessment, the Beck Anxiety Inventory (BAI) and the Beck Depression Inventory (BDI-II) were also performed with control subjects and BS patients at baseline and with BS patients at the six-month follow-up.

Postoperative delirium was assessed by trained clinical professionals twice daily while the patient was in the hospital, using the standardized Confusion Assessment Method and by review of their electronic medical records.

### 2.3 Magnetic resonance imaging

The impact of surgery on brain structure revealed with MRI was considered the main outcome of this study. Brain imaging scans were obtained using a 3.0-Tesla MR scanner (Siemens, Magnetom Prisma, Erlangen, Germany). As previously described,(Roy et al., 2022) two high-resolution T1-weighted images were acquired using a magnetization prepared rapid acquisition gradient-echo (MPRAGE) pulse sequence [repetition-time (TR) = 2200 ms; echo-time (TE) = 2.41 ms; inversion-time = 900 ms; flip-angle (FA) = 9°; matrix-size = 320 × 320; field-of-view (FOV) = 230 × 230 mm^2^; slice-thickness = 0.9 mm; number of slices = 192]. Proton-density (PD) and T2-weighted imaging (TR = 10,000 ms; TE1, TE2 = 12, 124 ms, FA = 130°; matrix size = 256 × 256; FOV = 230 × 230 mm^2^; slice-thickness = 3.5 mm; inter-slice gap = no) were performed simultaneously using a dual-echo turbo spin-echo pulse sequence, covering the entire brain in the axial plane.

High-resolution T1-weighted, PD-, and T2-weighted images were visually examined for any brain pathology (MRIcron) and assessed for motion-related or any other imaging artifacts before quantification of gray matter volume and T2-relaxation maps.

### 2.4 Calculation of gray matter volume and quantification of T2-relaxation

High-resolution T1-weighted image series were reoriented to remove any potential variations between the scans from head motion and averaged to increase the signal-to-noise ratio. The T1-weighted images were realigned in the space of first series by co-registering (FWHM, 3mm) all the images to the first scan of the first session. The averaged images were partition in gray and white matter, and cerebral spinal fluid tissue types, using the DARTEL toolbox(Ashburner, 2007), and created flow fields and a series of template images. The flow fields were used to normalize gray matter maps (modulated, resliced to 1 × 1 × 1 mm^3^) to Montreal Neurological Institute (MNI) space and smoothed using a Gaussian filter (Roy et al., 2020; Roy et al., 2021a).

Using PD and T2-weighted images, whole brain pixel-by-pixel T2-relaxation values were quantified and whole-brain T2-relaxation maps were generated with voxel intensity corresponded to the calculated T2-relaxation time. The average noise threshold outside the brain tissue was calculated from PD- and T2-weighted images and was used as a masking threshold to exclude non-brain areas. The same noise threshold was used for the PD and T2-weighted images for all subjects. A ceiling threshold of 500 ms was applied to all T2-relaxation maps during T2-relaxation calculation to restrict cerebrospinal fluid values. The T2-relaxation values were quantified using the equation below (Roy et al., 2021a; Roy et al., 2021b):


$$T_{2}=\frac{\left(T\,E_{2}-T\,E_{1}\right)}{\text{ln}\,\left(\frac{S\,I_{1}}{S\,I_{2}}\right)}$$

where TE_1_ and TE_2_ are the echo-times for PD and T2-weighted images, and SI_1_, SI_2_ represent PD and T2-weighted images signal intensities, respectively. T2-relaxation maps were normalized to the standard MNI space and smoothed using a Gaussian filter (8 mm).

### 2.5 Statistical methods

Statistical analysis was performed using IBM SPSS Statistics for Windows, Version 28.0 (Armonk, NY: IBM Corp) and the statistical parametric mapping (SPM12; Wellcome Department of Cognitive Neurology, UK). MATLAB-based (The MathWorks Inc, Natick, MA, USA) software was used to process and analyze MRI data, as previously described (Roy et al., 2020; Roy et al., 2021a; Roy et al., 2021b).

Differences between BS patients and controls characteristics were assessed using *X*^2^ or Fisher exact tests for categorical variables and independent samples *t* test or Mann Whitney U test for continuous variables, according to normality tests. Neurocognitive performance was compared between BS patients and controls using independent samples *t* test and within the BS group using paired samples *t* test.

Analysis of covariance was employed to assess differences in regional brain gray matter volume and brain tissue changes between BS patients at baseline and controls. Longitudinal brain changes within the BS group were assessed with paired samples *t* test. Brain clusters with significant gray matter volume and T2-relaxation value differences between groups were overlaid onto the normalized mean anatomical images for structural identification. Both analyses to assess differences in regional brain gray matter volume and brain tissue changes considered age and sex as covariates. False discovery rate was corrected for multiple comparison while accessing regional brain gray matter volume.

Statistical tests employed in the analysis were two-sided with a set p value for significance of < 0.05. Since this was a pilot study no previous sample size estimation was performed.

## 3 Results

### 3.1 Participants

A total of 19 patients scheduled for BS were included in this analysis [age, 54 (9) years; BMI, 40 (36,42) kg m^–2^] (Supplementary Figure 1). All patients completed baseline activities, and 15 patients completed the immediate MRI postoperative evaluations. No immediate postoperative complications were identified by the clinical teams. Five patients were lost at the six-month follow-up. For baseline assessment, we used 50 control subjects (age, 52 (6) years; BMI, 25 (24,27) kg m^–2^). Table 1 describes the baseline characteristics of the BS and control groups. Patients from the BS group lost an average of 22 (8) kg reflected in a BMI of 31 (29,33) kg m^–2^ at the six-month follow-up.

**TABLE 1:** Participants and controls characteristics.

Variables	Controls [*n* = 50]	Bariatric surgery group [*n* = 19]	*P value*
**Age, years, mean (SD)**	52 (6)	54 (9)	0.435
**Sex, female, *n* (%)**	24 (48)	14 (74)	0.055
**BMI, kg m-^2^, median (Q1, Q3)**	25.0 (23.6, 27.1)	39.5 (36.1, 41.8)	< 0.001
**Ethnicity, *n* (%)**			0.001
Not Hispanic or Latino	45 (90)	10 (53)	
Hispanic or Latino	5 (10)	9 (47)	
**Self-Reported Race, *n* (%)**			0.476
White	28 (56)	14 (74)	
Asian	13 (26)	2 (11)	
African American	4 (8)	1 (4)	
More than one	5 (10)	2 (11)	
**ASA, *n* (%)**	NA		NA
2		2 (11)	
3		16 (84)	
4		1 (5)	
**Surgery type, *n* (%)**	NA		NA
Laparoscopic Sleeve Gastrectomy		17 (89)	
Laparoscopic Roux-en-Y Gastric Bypass		2 (11)	
**Anesthesia time, minutes, mean (SD)**	NA	147 (32)	NA

ASA, American Society of Anesthesiologists; BMI, body mass index; N, number; NA, Not applicable; Q, quartile; SD, standard deviation.

### 3.2 Neurocognitive deterioration

**FIGURE 2:**
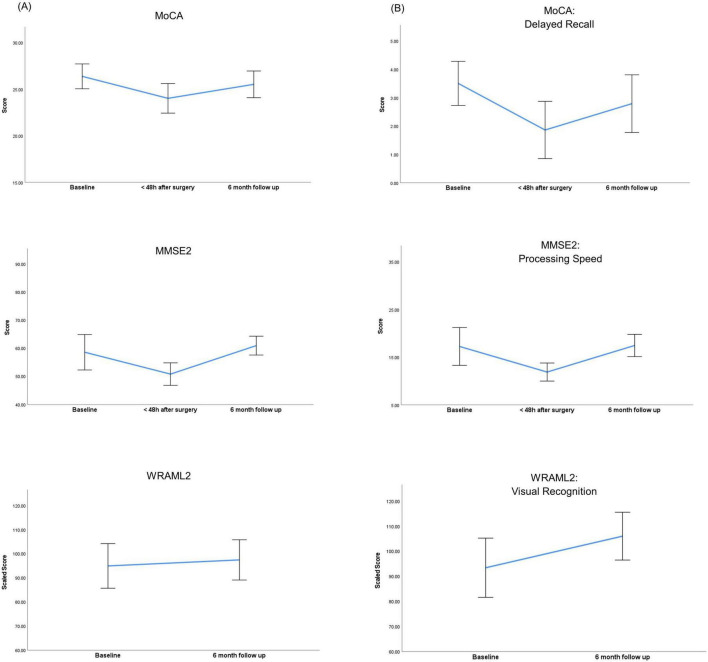
Perioperative trajectories of neurocognitive scores within the bariatric surgery group. **(A)** Neurocognitive variations of overall scores. **(B)** Neurocognitive variations in specific domains. MMSE2, Mini Mental State Examination 2nd edition; MoCA, Montreal Cognitive Assessment; WRAML2, Wide Range Assessment of Memory and Learning 2. Error bars represent the 95% confidence interval of the mean scores.

Compared with controls, neurocognitive assessment at baseline in the BS group revealed a significantly lower overall score (MoCA 26 vs 28 *P* = 0.017). All patients included in the analysis had 12 years or more of education. Bariatric surgery induced a significant decrease in cognitive function in the immediate postoperative period, reflected by lower global scores of MoCA (26 vs 24, *P* < 0.001) and MMSE2 (59 vs 51, *P* < 0.001) when compared with baseline assessment (Figure 2 and Table 2). Specific domains comprehending visuospatial, retrieval memory, and processing speed were the most affected (Figure 2). The Confusion Assessment Method was negative for all patients. There was no evidence that any patient developed postoperative delirium.

**TABLE 2:** Neurocognitive assessment between bariatric patients and controls and within bariatric surgery group.

Variables		Bariatric surgery			*P*-value (controls vs baseline)	*P*-value (baseline vs < 48h after surgery)	*P*-value (baseline vs six-month follow-up)
	Controls [*n* = 50]	Baseline [*n* = 19]	< 48h after surgery [*n* = 19]	Six-month follow-up [*n* = 14]			
**Global MoCA**	28 (2)	26 (3)	24 (3)	26 (3)	0.017	< 0.001	0.195
Visuospatial	5 (1)	4 (1)	4 (1)	4 (1)	0.042	0.008	0.263
Naming	3 (0)	3 (0)	3 (0)	3 (0)	0.331	0.331	1.000
Attention	6 (1)	5 (1)	5 (1)	5 (1)	0.220	0.408	0.720
Language	3 (1)	2 (1)	3 (1)	3 (1)	0.744	0.429	0.775
Abstraction	2 (0)	2 (1)	2 (1)	2 (0)	0.142	0.104	0.336
Delayed recall	4 (1)	3 (2)	2 (2)	3 (2)	0.128	<0.001	0.108
Orientation	6 (0)	6 (0)	6 (0)	6 (0)	1.000	1.000	1.000
**MMSE-2:EV**	NA	59 (11)	51 (7)	61 (6)	NA	< 0.001	0.298
Visuospatial		1 (0)	1 (0)	1 (0)		0.578	1.000
Naming		2 (0)	2 (0)	2 (0)		1.000	1.000
Attention		4 (2)	4 (2)	3 (2)		0.202	0.108
Language		6 (0)	6 (0)	6 (0)		1.000	1.000
Working Memory		3 (0)	3 (0)	3 (0)		0.331	0.165
Delayed recall		2 (1)	1 (1)	2 (1)		<0.001	0.290
Orientation		10 (0)	10 (0)	10 (0)		1.000	0.336
Verbal explicit learning / free recall		14 (5)	13 (4)	17 (4)		0.180	0.022
Processing Speed		17 (6)	13 (4)	17 (4)		<0.001	0.890
BV		15 (1)	14 (1)	15 (1)		<0.001	0.373
SV		28 (2)	27 (2)	27 (2)		0.025	0.050
**WRAML2**	NA	97 (18)	NA	99 (15)	NA	NA	0.459
Verbal memory		93 (14)		99 (11)			0.172
Visual memory		106 (14)		108 (15)			0.356
Attention/concentration		93 (17)		90 (21)			0.403
Working Memory		95 (15)		92 (17)			0.709
General Recognition		102 (23)		106 (13)			0.092
Verbal Recognition		97 (21)		103 (14)			< 0.001
Visual Recognition		104 (20)		114 (32)			0.482
**BAI**	2 (3)	12 (13)	NA	12 (13)	0.009	NA	0.701
**BDI II**	2 (3)	11 (9)	NA	8 (7)	< 0.001	NA	0.214

BAI, Beck Anxiety Inventory; BDI, Beck Depression Inventory; BV, brief version; EV, expanded version; MMSE2, Mini-Mental State Examination 2^nd^ edition; MoCA, Montreal Cognitive Assessment; NA, not applicable; SV, standard version; WRAML2, Wide Range Assessment memory and Learning 2. Values are presented as mean (standard deviation).

Compared with baseline, evaluation at six months demonstrated a recovery of cognitive function in all domains. Verbal memory domains comprehending explicit learning / free recall and recognition showed a significant improvement at six months when compared to baseline (14 vs 17, *P* = 0.022 and 97 vs 103, *P* < 0.001).

### 3.3 Gray matter volume changes

**FIGURE 3:**
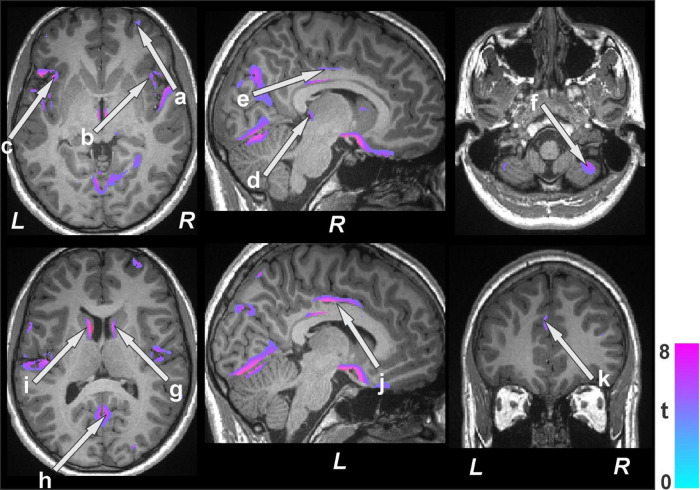
Brain regions with decreased gray matter volumes in bariatric surgery patients (*n* = 19) at baseline over controls (*n* = 50). These sites with reduced gray matter volumes included the cerebellar vermis, prefrontal (a) and ventral-medial prefrontal cortices, bilateral insula (b, c), thalamus (d), anterior (k), mid (e, j), posterior cingulate, cerebellar cortices (f), caudate (g, i), and lingual gyrus (h). All images are in neurological convention (L = left; R = right). Color bar indicates t-statistic values.

**FIGURE 4:**
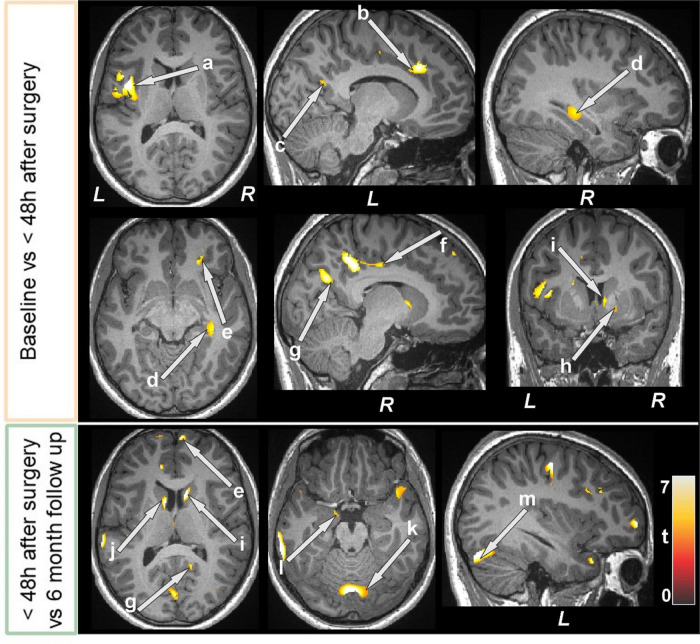
Increased gray matter volumes in bariatric surgery patients within 48 h of surgery (*n* = 15) over baseline with a further increase at the 6-month follow-up (*n* = 12) over < 48 h. These sites with higher gray matter volumes included the insula (a), anterior (b), mid (f), and posterior (c, g) cingulate, hippocampus (d), prefrontal cortices (e), putamen (h), caudate (i, k), lingual gyrus (h), amygdala (l), and cerebellar cortices (k, m). All images are in neurological convention (L = left; R = right). Color bar indicates t-statistic values.

Distinct regional brain tissue changes, such as decreased volume of gray matter, appeared in BS patients at baseline (*n* = 19) compared to healthy control subjects (*n* = 50) (Figure 3 and Table 3) while few sites were observed with greater or larger volumes of gray matter. The whole brain voxel-based comparison of BS patients at baseline with 48 h after surgery (*n* = 15) showed increased gray matter volumes in several regions (Figure 4 and Table 4). The left cerebellum is the only brain site that showed decreased gray matter volume after surgery. When comparing BS patients within 48 h of surgery and follow up at six months (*n* = 12) (Figure 4 and Table 4) we showed that most regions had increased volume, and some showed decreased gray matter volume.

**TABLE 3:** Regions with reduced gray matter volumes in bariatric surgery patients over control subjects.

Regions	Cluster size	Coordinates			*P-*value	Z score, T-score
		X	Y	Z		
Left Cerebellum	9828	−44	−77	−27	<0.001	7.36, 9.24
Left Cerebellum		−50	−51	−45		6.51, 7.77
Left Caudate	629	−9	4	15	<0.001	6.18, 7.24
Left Caudate		−8	11	11		5.63, 6.42
Left Caudate		−12	−3	20		4.51, 4.91
Left Mid Cingulate	3825	−11	−20	36	<0.001	6.10, 7.11
Left Mid Cingulate		−2	−11	29		4.99, 5.54
Left Mid Cingulate		−10	0	38		4.86, 5.37
Left Inferior Frontal Cortex	4001	−55	13	28	<0.001	5.62, 6.41
Left Inferior Frontal Cortex		−49	18	−4		5.15, 5.75
Left Inferior Frontal Cortex		−56	14	2		4.76, 5.23
Cerebellar Vermis	6968	−4	−83	−17	<0.001	5.28, 5.93
Cerebellar Vermis		1	−72	−12		5.02, 5.58
Left Occipital Lobe		−3	−64	0		4.20, 4.53
Right Cerebellum	4681	46	−43	−29	<0.001	5.16, 5.76
Right Cerebellum		27	−39	−53		5.00, 5.54
Right Cerebellum		37	−27	, −31		4.82, 5.31
Right Caudate	363	10	7	15	<0.001	4.80, 5.28
Right Caudate		12	−1	19		4.13, 4.44
Right Prefrontal Cortex	732	31	60	20	< 0.001	4.71, 5.16
Right Frontal Cortex		31	52	31		3.69, 3.91
Right Prefrontal Cortex		21	65	20		3.42, 3.59
Left Middle Frontal Cortex	367	−27	29	53	<0.001	4.55, 4.96
Left Middle Frontal Cortex		−29	16	61		4.10, 4.40
Left Prefrontal Cortex	247	−45	51	6	<0.001	4.46, 4.84
Right Thalamus	121	9	−32	2	<0.001	3.91, 4.17
Right Inferior Frontal Cortex	46	44	21	−13	< 0.001	3.65, 3.86
Left Medial Frontal Cortex	211	−2	39	24	<0.001	3.63, 3.84
Right Prefrontal Cortex	52	48	52	1	<0.001	3.53, 3.72
Right Cerebellum	33	11	−89	−26	<0.001	3.51, 3.70
Right Cerebellum	76	36	−84	−26	<0.001	3.47, 3.65
Right Anterior Cingulate	39	2	33	24	0.001	3.29, 3.44

**TABLE 4:** Brain volume change in bariatric surgery patients within 48 h after surgery over baseline, and at the 6-month follow-up over< 48 h after surgery.

Regions	Cluster size	Coordinates			*P* value	Z score, T-score
		X	Y	Z		
**< 48 h after surgery over baseline**						
Left Temporal Cortex	2908	−41	2	10	<0.001	5.03, 8.73
Left Temporal Cortex		−37	−2	18		4.47, 6.85
Left Insula		−39	−11	20		4.40, 6.67
Right Posterior Cingulate	1099	11	−45	40	<0.001	4.95, 8.44
Left Anterior Cingulate	411	−12	25	32	<0.001	4.89, 8.20
Right Caudate	245	7	4	2	<0.001	4.56, 7.14
Right Middle Cingulate	445	14	−19	37	<0.001	4.37, 6.58
Right Posterior Cingulate		16	−35	39		4.06, 5.76
Left Lingual Gyrus	244	−14	−59	19	<0.001	4.13, 5.94
Right Hippocampus	435	37	−29	−7	<0.001	4.10, 5.85
Left Amygdala	57	−33	−5	−22	<0.001	3.93, 5.45
Right Prefrontal Cortex	171	24	36	−10	<0.001	3.86, 5.30
Right Prefrontal Cortex		19	30	−15		3.64, 4.83
Right Putamen	71	18	6	−5	<0.001	3.84, 5.25
Left Prefrontal Cortex	29	−40	45	−6	<0.001	3.79, 5.13
Right Middle Cingulate	60	3	−11	44	<0.001	3.73, 5.00
Left Middle Cingulate	18	−11	6	44	<0.001	3.69, 4.92
Right Insula	12	36	−5	18	<0.001	3.64, 4.82
**6-month follow-up over< 48 h after surgery**						
Right Caudate	660	10	13	13	0.001	5.34, 12.28
Right Caudate		13	−3	21		3.74, 5.50
Right Caudate		16	−10	24		3.17, 4.18
Cerebellum	8755	0	−76	−19	<0.001	5.19, 11.33
Left Cerebellum		−12	−91	−31		5.04, 10.46
Right Cerebellum		32	−87	−27		4.93, 9.90
Right Anterior Cingulate	210	4	33	1	<0.001	4.94, 9.92
Left Caudate	634	−10	0	15	<0.001	4.75, 9.04
Left Caudate		−15	−13	23		3.55, 5.04
Right Prefrontal Cortex	469	33	59	4	<0.001	4.61, 8.42
Right Prefrontal Cortex		27	65	−6		3.43, 4.74
Left Anterior Cingulate	86	−4	33	1	<0.001	4.46, 7.81
Left Amygdala	1725	−15	0	−26	<0.001	4.45, 7.77
Left Superior Temporal Gyrus		−34	22	−33		4.36, 7.44
Left Superior Temporal Gyrus		−25	4	−35		4.01, 6.26
Left Prefrontal Cortex	702	−39	59	3	<0.001	4.41, 7.62
Left Prefrontal Cortex		−31	63	−2		4.14, 6.69
Left Prefrontal Cortex		−21	68	2		3.46, 4.81
Left Anterior Cingulate	71	−13	39	11	<0.001	4.32, 7.28
Right Prefrontal Cortex	210	6	68	11	<0.001	4.29, 7.20
Right Lingual Gyrus	352	16	−55	23	<0.001	4.27, 7.11
Right Lingual Gyrus		15	−54	14		4.02, 6.31
Left Parahippocampal Gyrus	586	−15	−32	−11	<0.001	4.02, 6.31
Left Parahippocampal Gyrus		−11	−38	−3		3.56, 5.05
Left Thalamus		−11	−30	−4		3.55, 5.03
Left Posterior Cingulate	155	−13	−55	1	0.001	3.82, 5.73
Left Posterior Cingulate		−9	−57	9		3.18, 4.21
Right Prefrontal Cortex	90	3	65	−4	<0.001	3.78, 5.60
Right Prefrontal Cortex		2	57	−8		3.77, 5.59
Right Lingual Gyrus	167	14	−67	−3	<0.001	3.75, 5.53
Left Lingual Gyrus	16	−16	−55	17	<0.001	3.69, 5.36
Thalamus	191	1	−8	1	0.001	3.58, 5.10
Left Thalamus		−4	−11	17		3.42, 4.72
Thalamus		0	−14	10		3.29, 4.43
Right Cerebellum	64	4	−62	−58	<0.001	3.56, 5.06
Left Insula	121	−42	−26	15	0.001	3.55, 5.04
Left Temporal Cortex		−45	−21	8		3.21, 4.26
Left Prefrontal Cortex	122	−2	54	−8	<0.001	3.51, 4.93
Left Prefrontal Cortex		−3	65	−2		3.49, 4.89
Left Cerebellum	188	−7	−65	−56	<0.001	3.50, 4.91
Right Cerebellum	21	7	−48	−25	<0.001	3.37, 4.61
Left Prefrontal Cortex	23	−14	69	11	<0.001	3.36, 4.59
Left Cerebellum	21	−25	−43	−44	<0.001	3.34, 4.54
Left Putamen	24	−22	−4	5	0.001	3.27, 4.39

### 3.4 Brain tissue changes

**FIGURE 5:**
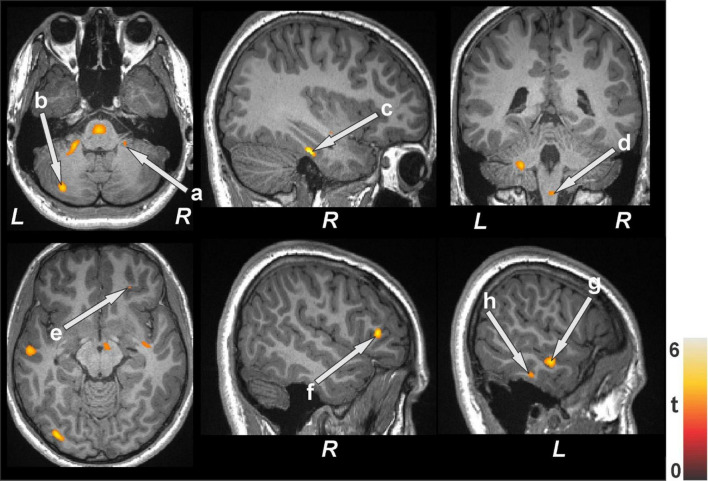
Increased T2-relaxation values in bariatric surgery patients at baseline (*n* = 18) over controls (*n* = 50). These sites with higher T2 relaxation values included the anterior cingulate, cerebellar cortices (a, b), parahippocampal gyrus (c), brain stem (d), prefrontal cortices (e, f), and temporal cortices (g, h). All images are in neurological convention (L = left; R = right). Color bar indicates t-statistic values.

Several brain areas showed increased T2-relaxation values in BS patients at baseline compared to control subjects (Figure 5 and Table 5). Few sites showed reduced T2 relaxation values in BS patients at baseline.

**TABLE 5:** Regions with higher T2-relaxation values in bariatric surgery patients over control subjects.

Regions	Cluster size	Coordinates			*P-*value	Z score, T-score
		X	Y	Z		
Right Parahippocampal Gyrus	313	36	−26	−28	<0.001	4.74, 5.21
Right Prefrontal Cortex	189	50	29	9	<0.001	4.14, 4.45
Right Prefrontal Cortex	21	6	29	−26	0.001	3.20, 3.35
Left Cerebellum	268	−31	−76	−43	<0.001	3.90, 4.16
Left Cerebellar Tonsil	1773	−20	−41	−41	<0.001	4.12, 4.42
Brain Stem		0	−29	−44		3.92, 4.18
Right Cerebellum		18	−36	−38		3.64, 3.85
Left Middle Temporal Cortex	401	−57	−15	−15	<0.001	4.03, 4.31
Left Inferior Temporal Cortex	139	−51	−36	−23	<0.001	3.44, 3.63
Left Inferior Temporal Cortex		−58	−31	−25		3.44, 3.62
Left Anterior Cingulate	4	−4	48	4	0.001	3.16, 3.30

**FIGURE 6:**
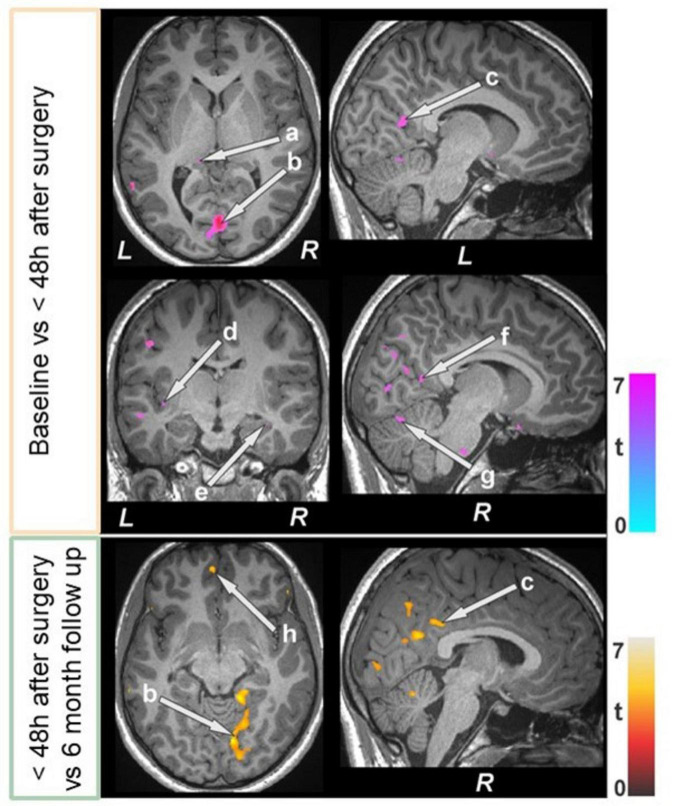
Bariatric surgery patients within 48 h of surgery (*n* = 15) showed reduced T2-relaxations over baseline, and some of regions showed increased values at 6 month follow up (*n* = 12). These sites with altered T2-relaxation values included the brain stem, basal forebrain, the thalamus (a), lingual gyrus (b), posterior cingulate (c, f), insula (d), hippocampus (e), cerebellum (g), and prefrontal cortices (h). All images are in neurological convention (L = left; R = right). Color bar indicates *t*-statistic values.

When comparing baseline and the immediate period after surgery, less than 48 h, reduced T2 relaxation values appeared in many areas of the brain (Figure 6 and Table 6). The bilateral caudate and right pallidum showed increased T2 relaxation values. Importantly, the T2 relaxation values at six months follow-up (compared to 48 h after surgery) increased in several regions (Figure 6 and Table 6). Whereas T2 relaxation values were decreased in the brain stem, left caudate, cerebellum, right prefrontal cortices, bilateral mid and right superior frontal cortices.

**TABLE 6:** T2-relaxation of bariatric surgery patients within 48 h after surgery over baseline, and at the 6-month follow-up over< 48 h after surgery.

Regions	Cluster size	Coordinates			*P-*value	Z score, T-score
		X	Y	Z		
**< 48 h after surgery over baseline**						
Left Lingual Gyrus	2564	2	−79	3	<0.00	4.29, 6.34
Right Lingual Gyrus		3	−81	14	1	3.77, 5.09
Right Posterior Cingulate		8	−66	15		3.76, 5.08
Brain Stem	690	2	−25	−42	<0.001	4.12, 5.92
Right Cerebellum	87	36	−47	−24	<0.001	3.98, 5.57
Right Posterior Cingulate	281	16	−60	15	<0.001	3.93, 5.46
Left Posterior Cingulate	525	−4	−59	14	0.001	3.65, 4.83
Right Posterior Cingulate		1	−57	26		3.53, 4.59
Right Posterior Cingulate		4	−64	23		3.22, 4.02
Right Cerebellum	77	39	−66	−36	<0.001	3.57, 4.68
Right Posterior Cingulate	62	9	−55	9	<0.001	3.51, 4.56
Brain Stem	46	−5	−45	−58	<0.001	3.38, 4.30
Right Insula	42	43	−25	18	< 0.001	3.38, 4.30
Left Thalamus	21	−13	−34	4	<0.001	3.36, 4.27
Right Lingual Gyrus	46	12	−92	−7	<0.001	3.32, 4.20
Left Cerebellum	14	−10	−44	−23	0.001	3.28, 4.12
Left Cerebellum	44	−9	−62	−15	0.001	3.26, 4.08
Left Basal Forebrain	40	−10	3	−12	0.001	3.18, 3.94
Left Insula	12	−38	−15	−3	0.001	3.18, 3.94
Right Hippocampus	2	35	−15	−17	0.001	3.13, 3.86
**6-month follow-up over< 48 h after surgery**						
Left Prefrontal Cortex	473	−2	51	−17	<0.001	4.69, 8.76
Right Parahippocampal Gyrus	4942	17	−39	−8	<0.001	4.28, 7.16
Right Lingual Gyrus		21	−85	−16		3.93, 6.03
Right Parahippocampal Gyrus		21	−58	−7		3.88, 5.89
Right Posterior Cingulate	427	5	−55	22	0.001	3.55, 5.02
Left Posterior Cingulate		0	−56	29		3.21, 4.26
Right Posterior Cingulate		4	−66	18		3.18, 4.21
Right Middle Cingulate	40	7	−26	44	<0.001	3.34, 4.55

## 4 Discussion

This study examined the cognitive trajectories of obese patients at risk for PND after BS with MRI-based imaging. We found that obesity was associated with lower cognitive scores, chronic brain tissue changes, and reduced gray matter volume when compared to healthy control subjects. Bariatric surgery and anesthesia induced significant structural brain tissue changes, both increased gray matter volume and reduced T2 relaxation values, and also lower cognitive function scores in the immediate postoperative period (less than 48 h). Six months after surgery, the volume changes observed in the gray matter rebounded or increased in most regions, while some areas showed reduction of volume; T2 relaxation values were increased in most of the regions, suggesting some improvement and recovery at the microstructural tissue level, which was also reflected in a rebound to baseline cognitive scores.

These results are consistent with previous imaging studies that showed abnormalities in brain morphology and function, including decreased volume of gray or white matter, diminished structural connectivity, increased white matter hyperintensity volume in vulnerable populations, and additional brain insult caused by surgery (Kant et al., 2023; Li et al., 2023). Brain remodulation induced by BS is also in line with previously described work focusing on long term assessment of obese patients submitted to bariatric surgery (Prehn et al., 2020; Saindane et al., 2020; Custers et al., 2024). These results are also consistent with both short and long-term cognitive assessment tests conducted on obese surgical patients and BS patients, both with and without MRI data (Alosco et al., 2014; Saindane et al., 2020). As one could expect, recent studies also underscore that the long-term impact of overall cognitive recovery after BS continues at 24 months because of continued weight loss, the primary goal of the procedure, which would presumably improve many areas of vascular and brain aging correlates associated with lower BMI (Custers et al., 2024). While the preponderous of studies are consistent with our results, to our knowledge, no other study combines cognitive assessment testing with the detailed structural MRI analysis we describe, nor do any of these other studies include data from the immediate postoperative period (within 48 h). Importantly, our study provides a more detailed imaging assessment of microstructural brain tissue and volumetric alterations and transformations immediately after surgery and delineation of affected brain regions. Further, unlike other studies, we also minimized bias from the intraoperative management with the deployment of a consistent anesthetic plan for all subjects using a processed EEG monitor while also maintaining tightly controlled mean arterial pressures through continuous noninvasive blood pressure monitoring. We ensured and safeguarded our outcomes to obviate any confounding variations in blood pressure or possible effects of anesthetic “overdose” or adjunct medications, such as benzodiazepines, ketamine, or dexmedetomidine.

Our study is consistent with the current and prevailing understanding of PND as a condition that has multiple and not mutually exclusive causes and mechanisms (Vacas et al., 2021). The underlying mechanisms involved in the abnormal cerebral vascular function and inflammatory effects on specific brain areas include: the reduction of volumes of the caudate, prefrontal cortices, and anterior cingulate, which are responsible for memory, learning, executive function, attention, and both emotional and social cognition. We observed higher T2-relaxation values, indicating a chronic pathologic condition that could have resulted from the comorbidities associated with obesity, such as neuronal and axonal loss, gliosis, and demyelination. Crucially, within 48 h of surgery, BS patients showed larger gray matter volume and reduced T2 relaxation values that are consistent with acute inflammatory tissue changes, possibly due to the surgical insult and are associated with increased glial activation. This comports with the prevailing view that the mechanism involved in the genesis of PND is neuroinflammation. The lower T2 relaxation values and gray matter volume could also be due to neural and axonal swelling, increased neural, glial cell size, and higher vascular density, which support metabolic demand, connective tissue, dendritic outgrowth, and synaptogenesis. Interestingly, at six months, the gray matter volume rebounded or increased in most regions. However, T2 relaxation values were increased in many areas of the brain, suggesting recovery of microstructural tissue changes and that these precede volumetric improvement of gray matter. This was reflected in a rebound in cognition but not in a significant improvement in anxiety and depression indexes for the BS patients. Although psychological comorbidities, such as depression and anxiety, are prevalent in obese patients and have been associated with PND development (Falk et al., 2022; Fu et al., 2023), in this study and for the population studied, it is unlikely that the surgical-induced brain changes were related to depression and anxiety symptoms. Depression and anxiety were evaluated in all patients, and we did not find changes between baseline and the six-month follow-up.

### 4.1 Limitations

There are several weaknesses in our study, one of which is the small sample size that may limit our analysis and results. Further studies are needed to validate our observations. As is the case with any longitudinal study and complexity of tests and timepoints, we did not manage to collect all sets of data for each patient recruited; of the 20 patients that were recruited for the study, only 12 completed the entire set of tests and MRI scans reported in our manuscript. The second weakness relates to the evaluation of BS populations for longer periods of time. A longer period might help us better understand further changes to the brain following BS. A recent study,(Custers et al., 2024) with a larger sample size, demonstrates a rebound of cognition, or even possibly better than baseline, after 24 months, albeit with much less radiological depth and fewer details than what we captured and presented in this manuscript: both in terms of testing, MRI equipment, scan methodology, analysis, and timepoints. A third major weakness of this study relates to the use of MoCA, MMSE2, and WRAML2 instruments to map cognitive trajectories in combination with imaging data and analysis without the inclusion and integration of genomic data, and other blood biomarkers, including inflammatory markers (McKay et al., 2022).

### 4.2 Future studies

Proteomics, lipidomics, and genomic analyses should be used in future studies. In addition, the integration of plasma and inflammatory markers would provide a more cogent and comprehensive demonstration of the underlying mechanisms and processes involved in PND. Future studies may also examine the extent to which cognitive trajectories might be altered with the newer glucagon-like peptide-1 receptor agonist drugs that are now widely used for weight management in order to better understand the underlying impact of surgery and anesthesia on the brain.

Obese patients exhibited impaired cognitive performance and altered brain structural integrity in cognitive regulatory areas. Patients undergoing BS surgery showed significant brain structural changes from baseline, as evidenced by altered gray matter volume and T2 relaxation values, indicative of brain injury. The integration of preoperative evaluation for early identification and deployment of best practices for brain health in patients at risk for cognitive injury, such as obese patients, can improve the overall cognitive trajectories of these patients. Advanced enhancement of overall health status has the potential to revert most of the injuries seen after surgery.

## Data Availability

The raw data supporting the conclusions of this article will be accessible and made available by the authors upon request.

## Supplementary material

Supplementary Figure 1 Participant flow study chart. MRI, Magnetic resonance imaging.
