# Supplementary figures and images for: Distinct brain and neurocognitive transformations after bariatric surgery: a pilot study
Source: Front Neurosci. 2024 Nov 5;18:1454284. doi: 10.3389/fnins.2024.1454284 (PMC11573770; doi:10.3389/fnins.2024.1454284)

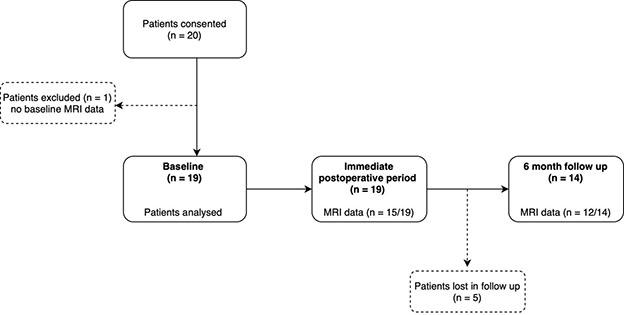

Supplement: Supplementary Figure 1 — Participant flow study chart. MRI, Magnetic resonance imaging. [file Image_1.jpeg]
